# The Esterase PfeE, the Achilles’ Heel in the Battle for Iron between *Pseudomonas aeruginosa* and *Escherichia coli*

**DOI:** 10.3390/ijms22062814

**Published:** 2021-03-10

**Authors:** Véronique Gasser, Laurianne Kuhn, Thibaut Hubert, Laurent Aussel, Philippe Hammann, Isabelle J. Schalk

**Affiliations:** 1 InnoVec, UMR7242, Université de Strasbourg, ESBS, Bld Sébastien Brant, F-67413 Illkirch, France; veronique.gasser@unistra.fr (V.G.); thibaut.hubert2@etu.unistra.fr (T.H.); 2 UMR7242, CNRS, ESBS, Bld Sébastien Brant, F-67413 Illkirch, France; 3 Plateforme Proteomique Strasbourg-Esplanade, Institut de Biologie Moléculaire et Cellulaire, CNRS, FR1589, 15 rue Descartes, F-67084 Strasbourg CEDEX, France; l.kuhn@ibmc-cnrs.unistra.fr (L.K.); p.hammann@ibmc-cnrs.unistra.fr (P.H.); 4 Laboratoire de Chimie Bactérienne, Institut de Microbiologie de la Méditerranée, Aix-Marseille University, CNRS, 13 900 Marseille, France; aussel@imm.cnrs.fr

**Keywords:** iron uptake, siderophore, enterobactin, iron homeostasis, *Pseudomonas aeruginosa*, outer membrane transporters, TonB, co-cultures

## Abstract

Bacteria access iron, a key nutrient, by producing siderophores or using siderophores produced by other microorganisms. The pathogen *Pseudomonas aeruginosa* produces two siderophores but is also able to pirate enterobactin (ENT), the siderophore produced by *Escherichia coli*. ENT-Fe complexes are imported across the outer membrane of *P. aeruginosa* by the two outer membrane transporters PfeA and PirA. Iron is released from ENT in the *P. aeruginosa* periplasm by hydrolysis of ENT by the esterase PfeE. We show here that *pfeE* gene deletion renders *P. aeruginosa* unable to grow in the presence of ENT because it is unable to access iron *via* this siderophore. Two-species co-cultures under iron-restricted conditions show that *P. aeruginosa* strongly represses the growth of *E. coli* as long it is able to produce its own siderophores. Both strains are present in similar proportions in the culture as long as the siderophore-deficient *P. aeruginosa* strain is able to use ENT produced by *E. coli* to access iron. If *pfeE* is deleted, *E. coli* has the upper hand in the culture and *P. aeruginosa* growth is repressed. Overall, these data show that PfeE is the Achilles’ heel of *P. aeruginosa* in communities with bacteria producing ENT.

## 1. Introduction

Iron is a vital nutrient involved in a wide range of enzymatic functions and biological processes and is essential for bacterial growth and virulence. The paradox of this key nutrient is its low bioavailability. Fe^3+^ is poorly soluble at neutral pH, with a limit of Fe^3+^ concentration in aqueous solutions of 10^−18^ M. Moreover, in mammals, iron is sequestered by proteins or heme to avoid Fe^2+^ toxicity (Fenton reaction) and maintain Fe^3+^ in a soluble form. Many bacteria overcome the problem of iron accessibility by producing siderophores, small organic compounds with an extremely high affinity for ferric iron [1].

Enterobactin (ENT), a triscatechol derivative of a cyclic triserine lactone, is an archetype in the field of siderophores. The chemistry, biology, and iron uptake by this siderophore have been extensively investigated over the last several decades, especially in *E. coli* [2,3]. ENT is produced by *Enterobacteriaceae* such as *Escherichia coli*, *Klebsiella pneumonia*, *Shigella flexneri*, and *Salmonella typhimurium* [4,5,6,7] and was thought to be unique to Gram-negative bacteria. However, the isolation of ENT from Gram-positive Streptomyces species [8] strongly suggests that the production of this siderophore may be wider than previously thought. ENT is synthesized in the bacterial cytoplasm by non-ribosomal peptide synthetases from chorismic acid [2].

**Figure 1:**
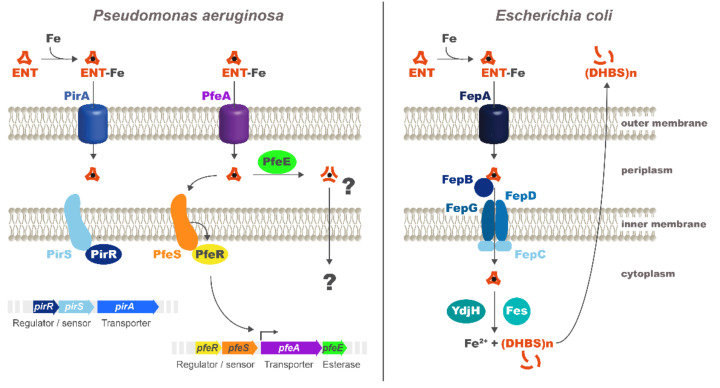
Iron uptake pathway *via* the siderophore enterobactin (ENT) in *P. aeruginosa* and *E. coli*. For more details, see the introduction.

ENT scavenges ferric iron in the bacterial environment with a Ka of 10^52^ M^−1^ [9]. Afterwards, ferric-ENT recapture by *E. coli* cells involves the TonB-dependent transporter (TBDT), FepA (Figure 1) [3,10]. This uptake involves the three conserved inner membrane proteins—TonB, ExbB, and ExbD—which transduce energy generated by the proton motive force of the inner membrane to the outer-membrane transporter, FepA [11]. In the periplasm, the ferri-ENT binds to the periplasmic binding protein, FepB [12], and is transported into the cytoplasm by the ABC transporter FepDGC (FepD and FepG forming the permease and FepC being the ATPase) [13,14,15]. In the cytoplasm, the esterase Fes hydrolyses ENT into three molecules of N-(2,3-dihydrobenzoyl) serine (DHBS), which are still able to chelate ferric iron [16,17,18]. Iron needs to be reduced by the NADPH-dependent reductase, YdjH, to be released from DHBS and transfered to cytoplasmic iron-binding proteins [19]. These gene sets (*fepA*, *fepB*, *fepDGC*, and *fes*) necessary to access to iron *via* the siderophore ENT in *E. coli* are present in many genomes (*Yersinia enterocolitica*, *Y. pestis*, *Y. pseudotuberculosis*, *Salmonella enterica*, *S. typhimurium*, and *Shigella* species), even if these bacteria are unable to produce ENT. Indeed, bacteria often use xenosiderophores (siderophores produced by other organisms) to access iron in a siderophore piracy strategy.

The opportunist pathogen *P. aeruginosa* also uses ENT as a xenosiderophore, but the molecular mechanisms involved are clearly different from those described for *E. coli*. Data from Poole *et al.* showed, at the beginning of the 1990s, that there are at least two uptake systems for ferri-ENT in *P. aeruginosa*: one of higher affinity, which is specifically inducible by ENT under iron-limiting conditions and involves the outer-membrane transporter, PfeA, and a second of lower affinity and independent of ENT for induction [20,21]. This notion was reinvestigated and the data clearly showed that the TBDT involved in high-affinity uptake is PfeA, that the transcription of *pfeA* gene is induced by ENT [21,22,23,24], and that the low-affinity TBDT is PirA [23]. Next to the *pfeA* gene on the chromosome is the *pfeE* gene, encoding a periplasmic esterase that acts by hydrolyzing ferri-ENT to promote iron release [25]. In *P. aeruginosa*, ENT releases iron in the bacterial periplasm and does not enter the cytoplasm at any time [25]. A similar mechanism has also been described for *Campylobacter*, in which a periplasmic trilactone esterase, Cee, also hydrolyses ferri-ENT in the bacterial periplasm [26]. Moreover, PfeA and PfeE expression is induced by the presence of ENT in the bacterial environment and a two-component system, PfeS/PfeR, with PfeS being the inner membrane sensor that detects the presence of ENT-Fe in the bacterial periplasm and PfeR the transcriptional regulator [22]. According to the *P. aeruginosa* genome, a similar PirS/PirR two-component system also regulates the transcription of PirA [27].

In addition to the ability to access iron *via* ENT, *P. aeruginosa* is able to produce two siderophores—pyoverdine (PVD) and pyochelin (PCH) [28]—and is also able to use many other xenosiderophores. For each of these siderophores or xenosiderophores used by the pathogen, *P. aeruginosa* possesses in its genome, genes encoding for a specific TBDT involved in the capture and uptake across the outer membrane of the ferric forms of these chelators and proteins involved in the mechanisms of iron release. Table S1 in the Supplementary Materials summarizes all the known iron uptake pathways used by *P. aeruginosa* PAO1.

Here, we further investigated iron uptake by ENT in *P. aeruginosa*. We show that ENT-Fe apparently interacts with PfeS, the sensor of the PfeS/PfeR two-component system, before its hydrolysis by PfeE. Moreover, in the absence of the sensor, PfeS, the two proteins, PfeA and PfeE, are constitutively expressed. Grow assays under iron restricted conditions showed that *P. aeruginosa* is unable to grow in the presence of ENT if *pfeE* is deleted. Finally, two-species co-cultures between *P. aeruginosa* and *E. coli* highlight the key and unique role PfeE plays in the ability of *P. aeruginosa* to grow in the presence of *E. coli* producing ENT, especially when *P. aeruginosa* is unable to produce its own siderophores.

## 2. Results

### 2.1. Insights on the Variation of pfeA and pfeE Trancription

**Figure 2:**
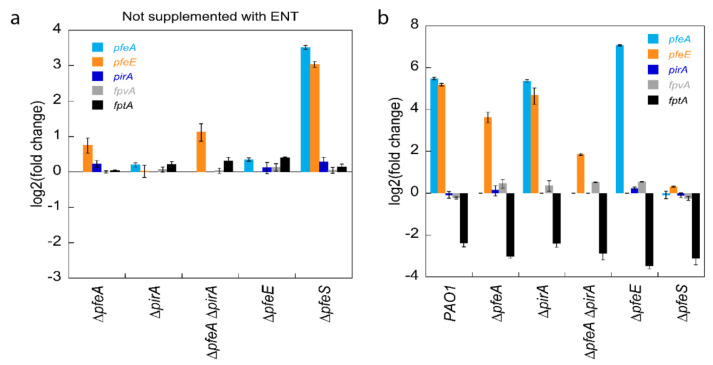
Analysis of changes in the transcription of the *pfeA, pfeE, pirA, fpvA* and *fptA* genes. RT-qPCR was performed on RNA isolated from *P. aeruginosa* PAO1 cells and the corresponding ∆*pfeA*, ∆*pirA*, ∆*pfeA*∆*pirA*, ∆*pfeE*, and ∆*pfeS* mutants, grown for 8 h in CAA medium. The results in panel (**a**) are given as the ratio between the values obtained for the various mutants (∆*pfeA*, ∆*pirA*, ∆*pfeA*∆*pirA*, ∆*pfeE* and ∆*pfeS*) over those obtained for PAO1, all grown in the absence of ENT. *pfeA* and *pirA* encode TBDTs involved in ENT-Fe uptake [21,23], *fpvA*, the TBDT of PVD-Fe [30], and *fptA* of PCH-Fe [31] (Table S1 in the Supplementary Materials). The results in panel (**b**) are given as the ratio between the values obtained for the various strains in the presence of ENT over those obtained for the same strains in the absence of ENT. For both panels (**a**,**b**), the data are normalized relative to the reference gene *uvrD* and are representative of three independent experiments performed in triplicate (*n* = 3).

Transcription of the *pfeA* and *pfeE* genes is induced by the PfeS/PfeR two-component system in the presence of ENT [29]. We used RT-qPCR to evaluate the importance of the proteins PfeA, PirA, PfeE, and PfeS in the regulation of the transcription of the *pfeA* and *pfeE* genes. Deletion mutants of the *pfeA*, *pirA*, *pfeE*, and *pfeS* genes (Table S2) were grown under iron-restricted conditions in the absence of ENT and the transcription levels of the *pfeA*, *pfeE*, and *pirA* genes followed (Figure 2a). *pfeS* deletion had an effect on the transcription of *pfeA* and *pfeE*, leading to log_2_ fold differences of 3.5 and 3.0, respectively, relative to their level of transcription in PAO1. In the absence of PfeS, PfeR probably becomes active because it is no longer associated with its inner-membrane sensor and, consequently, the regulator induces the transcription of the genes of the ENT pathway. The deletion of *pfeA* and of both *pfeA* and *pirA* had a small effect on the transcription of *pfeE*; in these two mutants, PfeR seems to be slightly more active via an unknown mechanism.

The experiment was repeated with 10 µM ENT in the growth media and we compared the level of transcription for each strain between cultures in the presence of ENT and those in its absence (Figure 2b). In PAO1, as previously described [24,29,32,33], the presence of 10 µM ENT in the growth medium induced the transcription of *pfeA* and *pfeE*, with a log_2_ fold change of 5.5 and 5.2, respectively. As discussed above, the absence of the sensor PfeS of the two-component system (∆*pfeS* strain) resulted in the induction of *pfeA* and *pfeE* gene transcription in the absence of ENT (Figure 2a). The addition of ENT to the growth media of this mutant had no additional effect on the transcription of these two genes (Figure 2b). Growth of the ∆*pfeA* strain in the presence of ENT resulted in the induction of *pfeE* transcription that was of lower intensity, as in PAO1 (log2 fold change of 3.6), probably because ENT-Fe cannot enter anymore in the *P. aeruginosa* periplasm by PfeA but only by PirA. *pfeA* and *pfeE* transcription in the ∆*pirA* mutant was similar as in PAO1, probably because all ENT-Fe is transported efficiently by PfeA and the uptake *via* PirA is less essential. The activation of *pfeE* transcription was very low for the double *pfeA* and *pirA* deletion mutant (∆*pfeA*∆*pirA*) (log_2_ fold change of 1.8), because there was no more uptake of ENT-Fe across the outer membrane. On the contrary, the presence of ENT in the growth media of ∆*pfeE* led to the strong induction of *pfeA* transcription (log2 of 7.1), indicating that ENT-Fe hydrolysis is not necessary for its interaction with PfeS of the two-component system. In the absence of PfeE, ferri-ENT was no longer hydrolyzed, probably accumulating in the bacterial periplasm and explaining the strong stimulation of *pfeA* transcription observed in the ∆*pfeE* strain. This observation is consistent with previous data showing that TCV-Fe (a non-hydrolysable tris-catechol iron chelator) is also able to induce *pfeA* and *pfeE* transcription with a higher efficiency than ENT-Fe [24]. Finally, *pirA* transcription was not activated by the presence of ENT in any of the strains or conditions tested, indicating that ENT-Fe is unable to interact with the sensor PirS of the PirS/PirR two-component system.

In conclusion, ENT-Fe needs to be transported across the *P. aeruginosa* outer membrane by either PfeA or PirA to activate the PfeS/PfeR two-component system. Once inside the bacterial periplasm, ENT-Fe interacts with the PfeS sensor to activate the transcription of the *pfeA* and *pfeE* genes before its hydrolysis by PfeE. PirA imports ENT-Fe across the outer membrane, but ENT-Fe is unable to activate the transcription of the *pirA* gene, which indicates that ENT-Fe is unable to interact with the two-component PirS/PirR system. Finally, PfeR is constitutively active in a *pfeS* deletion mutant, even in the absence of ENT.

### 2.2. Insights on the Mechanisms of Repression of PCH Pathway Transcription in the Presence of ENT

We previously showed that 10 µM ENT in the growth medium of *P. aeruginosa* cells also represses transcription of the genes encoding for the proteins involved in iron acquisition by the siderophore PCH (PCH pathway) produced by *P. aeruginosa* [24,32,33]. Here, we observed the repression of *fptA* transcription (TBDT of PCH-Fe) by RT-qPCR, with a log_2_ fold change between 2.3 and 3.5 for PAO1 and all mutants tested (Figure 2b), indicating that ENT-Fe does not need to enter *P. aeruginosa* cells by PfeA or PirA or interact somehow with the PfeS/PfeR two-component system to repress the endogenous PCH pathway. Such repression occurred only when ENT was present in the growth media and was completely independent of the expression of PfeA, PfeE, PfeS, and PfeR. The observed decrease in transcription of *fptA* and of the genes of the PCH locus [24,32,33] is assuredly a consequence of the competition for iron between ENT and PCH in the bacterial environment, resulting in less PCH-Fe being imported by FptA to activate the positive transcriptional regulator, PchR [34,35]. There was no effect on the transcription of *fpvA*, the TBDT of PVD-Fe, the other siderophore produced by *P. aeruginosa*.

### 2.3. PfeE Is an Irreplaceable Enzyme to Access Iron via ENT

**Figure 3:**
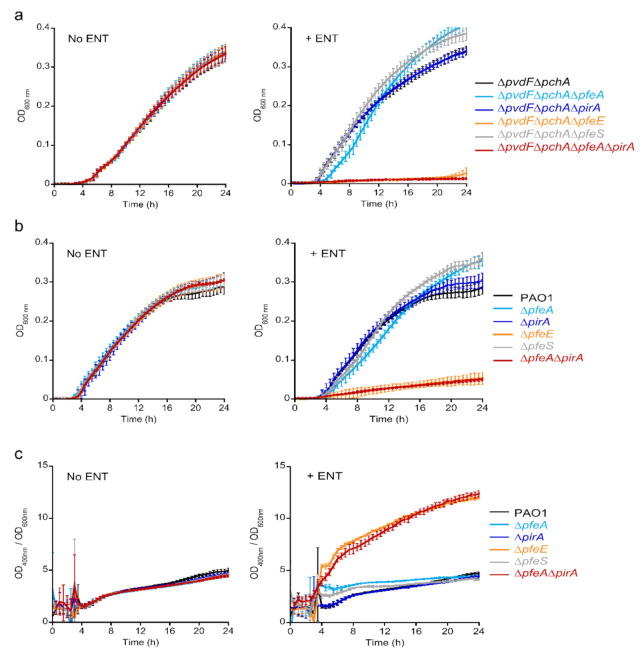
Growth of various *P. aeruginosa* mutants with or without 10 µM ENT in the CAA medium. In (**a**), the strains used are unable to produce the siderophores PVD and PCH: ∆*pvdF*∆*pchA* strain and its corresponding ∆*pvdF*∆*pchA*∆*pfeA*, ∆*pvdF*∆*pchA*∆*pirA*, ∆*pvdF*∆*pchA*∆*pfeA*∆*pirA*, ∆*pvdF*∆*pchA*∆*pfeE* and ∆*pvdF*∆*pchA*∆*pfeS* mutants. In (**b**), the strains are able to produce PVD and PCH; the *pfeA*, *pirA*, *pfeE* and *pfeS* mutations were carried out in PAO1. In (**a**,**b**), the strains were grown in the iron-restricted CAA medium with or without 10 µM ENT at 30 °C. Growth was followed by monitoring optical density (OD) at 600 nm. (**c**) PVD production during growth of PAO1, ∆*pfeA*, ∆*pirA*, ∆*pfeE*, ∆*pfeS*, and ∆*pfeA*∆*pirA* strains in CAA medium at 30 °C with or without 10 µM ENT. PVD production was followed by monitoring the characteristic absorbance at 400 nm of this siderophore [37]. Data are presented as the ratio between the OD at 400 nm and the OD at 600 nm. All data, panels (**a**–**c**), are the means of three independent experiments.

According to the literature, ENT-Fe complexes can be transported across the outer membrane by two TBDTs, PfeA and PirA [21,23,33]. Then, the siderophore-Fe complex is hydrolyzed by PfeE to facilitate iron release [25]. We evaluated the relative importance of the proteins encoded by the *pfeA*, *pirA*, and *pfeE* genes in the ability of *P. aeruginosa* cells to access iron in the presence of ENT by carrying out growth assays under iron-restricted conditions with *P. aeruginosa* strains deleted for at least one of these genes in (i) a PVD and PCH-deficient background (∆*pvdF*∆*pchA*, Table S2) to avoid any iron uptake by PVD and PCH, the two siderophores produced by *P. aeruginosa* (Figure 3a), and (ii) a wild-type background (Figure 3b). The growth assays were carried out in CAA medium (containing approximately 20 nM iron, [36]), with or without 10 µM ENT. As ENT is a very strong iron chelator (Ka = 10^52^ M^−1^, [9]), all iron is chelated by ENT under these growth conditions, resulting in ENT-Fe being the only iron source.

As previously described, the addition of ENT did not inhibit the growth of the *pfeA* and *pirA* single mutants [23] in either background (strains producing or not producing the siderophores PVD and PCH) (Figure 3a,b). In the ∆*pvdF*∆*pchA* background (strain unable to produce PVD and PCH), we observed complete growth inhibition with the double *pirA* and *pfeA* mutant, indicating that both of these transporters are involved in iron acquisition by ENT and that one can replace the other if it is absent, as described previously [23]. We also observed complete growth inhibition with the *pfeE* mutant in the ∆*pvdF*∆*pchA* background (∆*pvdF*∆*pchA*∆*pfeE* strain), confirming the key role of this esterase in iron acquisition by ENT: if ENT cannot be hydrolyzed by this enzyme, iron remains complexed with it in the bacterial periplasm and does not become accessible to the bacteria. In the PAO1 background, we observed strong growth inhibition with the *pfeE* mutation and the double *pfeA* and *pirA* mutation (strains ∆*pfeE* and ∆*pfeA*∆*pirA*), but with residual growth. Such residual growth is likely due to the high production of the siderophore PVD that is observed in these mutants (Figure 3c). We were unable to monitor PCH production, even after extraction from the growth media, because of the overlap of the absorbance spectra of PCH and ENT.

Deletion of the inner membrane sensor *pfeS* had no effect on bacterial growth (Figure 3a,b), which is consistent with the RT-qPCR data above, showing that the *pfeA* and *pfeE* genes are transcribed and the corresponding proteins are certainly expressed in this mutant. There was no increase in PVD production by the ∆*pfeS* strain compared to PAO1 (Figure 3c), indicating that the PVD pathway is not involved in iron uptake in this mutant.

The levels of PVD production were highly similar between the PAO1, ∆*pfeA*, ∆*pirA*, and ∆*pfeS* strains in the absence or presence of ENT; the addition of ENT did not stimulate the production of PVD (panels c). Stimulation occurred only for the ∆*pfeE* and ∆*pfeA*∆*pirA* mutants, which are unable to access iron chelated by ENT. *P. aeruginosa* adapted by increasing its PVD production. This adaptation does not appear to be essential for the growth of any of the other mutants, the pathogen being able to access iron either via ENT or the lower levels of PVD produced.

In conclusion, ENT-Fe complexes can be imported across the outer membranes of *P. aeruginosa* by the two TBDTs, PfeA and PirA. If one is absent, the other is able to take over uptake. On the contrary, PfeE cannot be replaced; in its absence, bacteria are unable to access iron from ENT. Finally, a *pfeS* mutant is able to access iron *via* ENT like PAO1, because both PfeA and PfeE are expressed, as in the wild type strain.

### 2.4. Co-Cultures between P. aeruginosa and E. coli: The Impact of ENT

**Figure 4:**
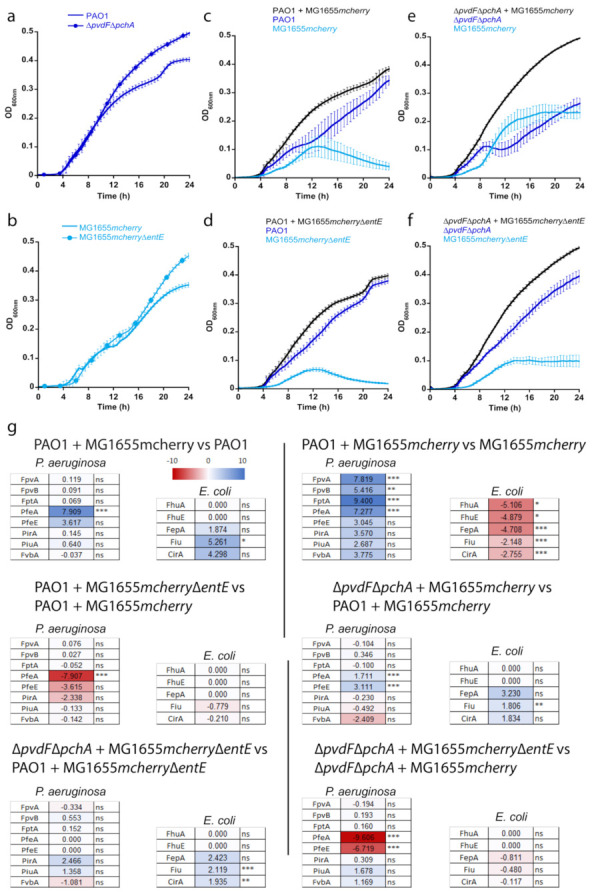
Co-cultures between *P. aeruginosa* and *E. coli*. All cultures were carried out in CAA medium supplemented with 0.6% glycerol at 30 °C. (**a**) Growth of *P. aeruginosa* strains producing, or not, PVD and PCH (PAO1 and ∆*pvdF*∆*pchA*). Both strains were grown alone. (**b**) Growth of *E. coli* producing, or not, ENT (MG1655*mcherry* or MG1655*mcherry*∆*entE*). Both strains were grown alone. (**c**–**f**) Co-cultures between *P. aeruginosa* strains producing, or not, PVD and PCH (PAO1 and ∆*pvdF*∆*pchA*, respectively) with *E. coli* producing, or not, ENT ((MG1655*mcherry* or MG1655*mcherry*∆*entE*, respectively). For the co-cultures, both strains were mixed at an OD of 0.005 and the bacterial growth followed by monitoring the OD at 600 nm. MG1655*mcherry* and MG1655*mcherry*∆*entE* both carry a plasmid encoding mCherry and the fluorescence of this protein was followed at 610 nm (excitation: 570 nm) (Figure S1 in Supplementary Materials). These values at 610 nm were used to identify the portion of the OD signal at 600 nm in the co-cultures due to *E. coli* and that due to *P. aeruginosa*. All data (panels (**a**–**f**)) are the means of three independent experiments. Figure S1 also shows the production of PVD by PAO1 in the various co-cultures. (**g**). Differential proteomic analyses were performed in parallel on the *P. aeruginosa* and *E. coli* proteomes for the cultures and co-cultures presented in panels (**a**–**f**). Volcano plots are shown in Figure S2 for *P. aeruginosa* and in Figure S3 for *E. coli*. Panel (**g**) shows the heat maps of these differential proteomic analyses for some TBDTs involved in iron-uptake in *P. aeruginosa* and in *E. coli*. For *P. aeruginosa*, FpvA and FpvB are the outer membrane transporters of ferri-PVD [30,38], FptA of ferri-PCH [31], PfeA and PirA of ferri-ENT [21,23], PiuA [39] and FvbA [40] of ferri-catechol-type siderophores, and, finally, PfeE is the esterase of the ENT pathway [25]. For *E. coli*, FepA is the outer membrane transporter of ferri-ENT, Fiu and CirA of other ferri-catechol-siderophores, and FhuA and FhuE of hydroxamate siderophores [41]. The darker the shade of blue, the higher the expression of the protein, and the darker the shade of red, the more the expression of the protein is repressed * *p* < 0.05, ** *p* < 0.01, *** *p* < 0.001.

We investigated the importance of ENT in co-cultures between *P. aeruginosa* and *E. coli* strains. We studied this bi-species system in planktonic culture using an iron-restricted medium in which both strains show very similar growth rates (Figure 4a,b). We first grew *P. aeruginosa*, able, or not, to produce its own siderophores PVD and PCH (PAO1 or ∆*pvdF*∆*pchA* strains) in the presence of *E. coli* strains, able, or not, to produce ENT (MG1655*mcherry* or MG1655*mcherry*∆*entE* strains), which resulted in four combinations of cultures (Figure 4c–f). Both strains of *E. coli* carry a plasmid harboring the gene encoding the fluorescent protein mCherry, used to differentiate *E. coli* from *P. aeruginosa* in the co-cultures. The OD was monitored at 600 nm and the mCherry fluorescence at 610 nm (excitation 570 nm) for each co-culture. The two strains of *E. coli* were also grown alone to be able to relate the intensity of mCherry fluorescence to the OD at 600 nm of *E. coli* throughout the co-culture (Figure S1). These values were used to identify the portion of the OD signal at 600 nm in the co-cultures due to *E. coli* and *P. aeruginosa*. The production of the siderophore PVD by *P. aeruginosa* strains in the various co-cultures was followed by monitoring the characteristic absorbance of PVD at 400 nm [37] (Figure S1). PCH production by *P. aeruginosa* and ENT production by MG1655*mcherry* could not be monitored directly in the bacterial cultures because the corresponding absorbances were too low and also slightly overlapping.

When *P. aeruginosa* was able to produce its own siderophores—PVD and PCH—it largely outgrew *E. coli* in co-cultures (*E. coli* producing or not producing ENT, MG1655*mcherry* or MG1655*mcherry*∆*entE* strains). However, when the ∆*pvdF*∆*pchA* strain was co-cultured with the strain of *E. coli* capable of producing ENT (MG1655*mcherry*), the two bacterial strains were able to co-exist, showing relatively similar ODs. Finally, when neither of the two strains produced siderophores (∆*pvdF*∆*pchA* and MG1655*mcherry*∆*entE*), *P. aeruginosa* once again outgrew *E. coli*.

**Figure 5:**
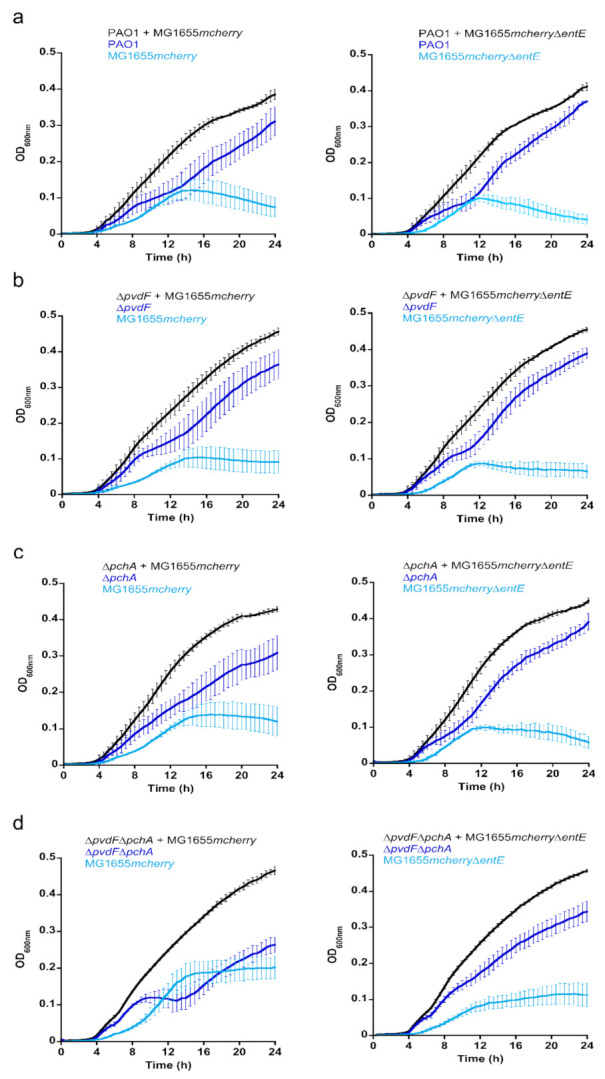
**Co-cultures between *P. aeruginosa* able to produce either PVD or PCH and *E. coli*.** Co-cultures of *P. aeruginosa* strains producing, or not, either PVD, PCH, or both siderophores (PAO1 (**a**), ∆*pvdF*(**b**), ∆pchA (**c**) and ∆*pvdF*∆*pchA*(**d**)) with *E. coli* producing, or not, ENT (MG1655*mcherry* or MG1655*mcherry*∆*entE*, respectively). All cultures were carried out in CAA medium supplemented with 0.6% glycerol at 30 °C. For the co-cultures, both strains were mixed at an OD of 0.005 and the bacterial growth monitored by following the OD at 600 nm. MG1655*mcherry* and MG1655*mcherry*∆*entE* both carry a plasmid encoding mCherry and the fluorescence of this protein was followed at 610 nm (excitation: 570 nm). These values at 610 nm were used to identify the portion of the OD signal at 600 nm in the co-cultures due to *E. coli* and *P. aeruginosa*. All the data are the means of three independent experiments.

We also investigated the importance of each siderophore produced by *P. aeruginosa*, PVD and PCH, in the competition in the co-cultures (Figure 5). Strains unable to produce either PVD or PCH were grown in the presence of MG1655*mcherry* or MG1655*mcherry*∆*entE*. As long as one of the siderophores, PVD or PCH, was produced by *P. aeruginosa*, it made up the majority in the co-cultures and *E. coli* growth remained repressed. When *P. aeruginosa* was unable to produce either of its siderophores, the growth of *E. coli* was less repressed and both bacterial species were able to grow in similar proportions.

Overall, these data show that the ability to produce ENT is an advantage for *E. coli* only if *P. aeruginosa* is unable to produce both of its own siderophores—PVD and PCH. Under all the other conditions tested, *P. aeruginosa* repressed the growth of *E. coli*.

### 2.5. Phenotypic Adaptation of P. aeruginosa and E. coli When Grown Together

We used a differential proteomic approach to compare the proteomes of *P. aeruginosa* and *E. coli* in the various cultures and co-cultures presented in Figure 4. The volcano plots of these analyses are presented in Figure S2 for *P. aeruginosa* and Figure S3 for *E. coli*. The heat maps of the outer membrane transporters of PVD (FpvA and FpvB, [30,38]), PCH (FptA, [31]), ENT (PfeA and PirA, [21,23]), two other catechol-siderophore outer-membrane transporters (FvbA and PiuA, [39,40]), and the esterase PfeE in *P. aeruginosa* [25] are presented in Figure 4g.

The presence of the ENT-producing MG1655*mcherry* induced strong production of PfeA and PfeE in *P. aeruginosa* PAO1 relative to the culture of *P. aeruginosa* alone in CAA (Figure 4g, PAO1 + MG1655*mcherry* vs. PAO1). The expression of FpvA, FpvB, and FptA was not modified, nor the expression of the other proteins of the PVD and PCH pathways (Figure S2). Differential proteomic analyses of the co-culture between MG1655*mcherry* and *P. aeruginosa* PAO1 compared to the culture of *E. coli* alone clearly show that FpvA, FpvB, FptA, were highly expressed (Figure 4g heat map of PAO1 + MG1655*mcherry* vs. MG1655*mcherry*) as well as all proteins of the PVD and PCH pathways (Figure S2) and PirA, PiuA, and FvbA, the TBDTs involved in iron acquisition by catechol siderophores. In the co-culture, *P. aeruginosa* PAO1 with MG1655*mcherry*, the catechol siderophore TBDTs FepA, Fiu, and CirA are expressed in *E. coli*, but not FhuA and FhuE, the ferrichrome and desferioxamine TBDTs.

When *E. coli* did not produce ENT (MG1655*mcherry*∆*entE*), PfeA was no longer expressed in *P. aeruginosa* PAO1 cells (Figure 4g heat map of PAO1 + MG1655*mcherry*∆*entE* vs. PAO1 + MG1655*mcherry*). There was slight repression of PirA and PfeE, but FpvA, FpvB, and FptA production were not modified. The expression of *E. coli* TBDTs was not modified compared to the co-culture between MG1655*mcherry* and PAO1 (Figure 4g heat map PAO1 + MG1655*mcherry*∆*entE* vs. PAO1 + MG1655*mcherry*).

When *P. aeruginosa* was unable to produce PVD and PCH and in the presence of *E. coli* producing ENT, the expression of the various proteins of the PVD and PCH pathways was not affected (Figure S2); these proteins all showed approximately the same level of production as in the co-culture between PAO1 and MG1655*mcherry* (Figure 4, ∆*pvdF*∆*pchA* + MG1655*mcherry* vs. PAO1 + MG1655*mcherry*). Moreover, in these conditions, PfeA and PfeE expression was slightly induced, indicating that *P. aeruginosa* probably uses more ENT, as in the condition of co-cultures presented in panel 4c. *E. coli*, in these co-culture conditions, increased the expression of FepA, Fiu, and CirA (Figure 4, ∆*pvdF*∆*pchA* + MG1655*mcherry* vs. PAO1 + MG1655*mcherry*).

Finally, when *P. aeruginosa* was unable to produce PVD or PCH and *E. coli* was unable to produce ENT, the expression of PfeA and PfeE dropped sharply relative to the culture between ∆*pvdF*∆*pchA* and MG1655*mcherry* (Figure 4g, ∆*pvdF*∆*pchA* + MG1655*mcherry*∆*entE* vs. ∆*pvdF*∆*pchA* + MG1655*mcherry*).

In conclusion, *P. aeruginosa* adapts its phenotype to the ability of *E. coli* to produce, or not ENT, by expressing, or not, PfeA and PfeE; in the presence of ENT-producing *E. coli*, *P. aeruginosa* induces the expression of the PfeA and PfeE proteins.

### 2.6. PfeE: The Achilles’ Heel of P. aeruginosa in Co-Cultures with E. coli

**Figure 6:**
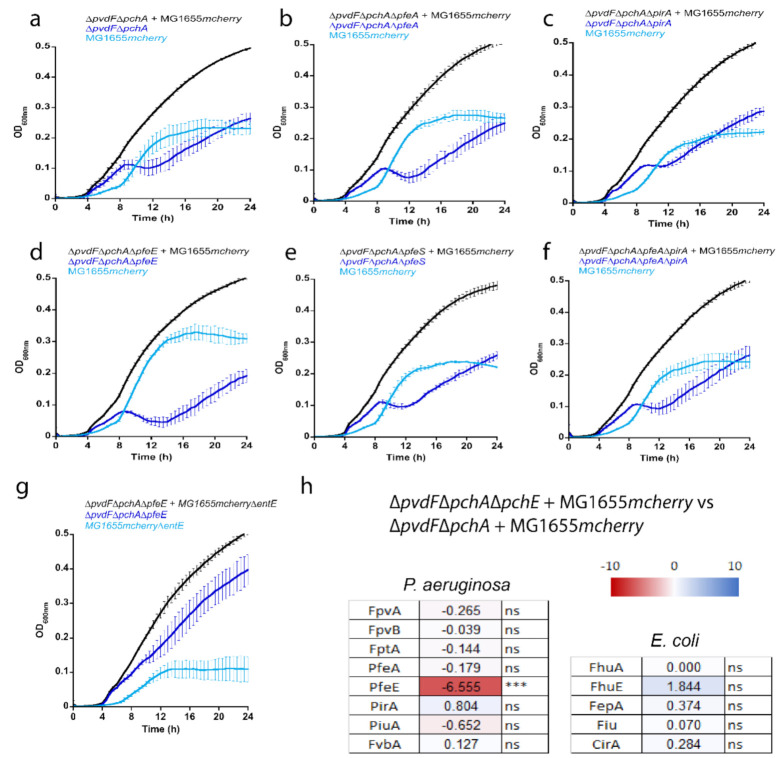
Co-cultures between *E. coli* MG1655*mcherry* and mutants of the ENT pathway of *P. aeruginosa*. (**a**–**f**) Co-cultures between *E. coli* MG1655*mcherry* and *P. aeruginosa* strains unable to produce PVD and PCH (∆*pvdF*∆*pchA*) and deleted for one of the genes of the ENT pathway (*pfeA*, *pirA*, *pfeE*, or *pfeS*). (**g**) Growth of *E. coli* MG1655*mcherry*∆*entE* and ∆*pvdF*∆*pchA*∆*pfeE*. All co-cultures in panels (**a**–**g**) were carried out in CAA medium supplemented with 0.6% glycerol at 30 °C and both strains were mixed at an OD_600 nm_ of 0.005. Bacterial growth was followed by monitoring the OD at 600 nm. As in Figure 4 and Figure 5, MG1655*mcherry* carries a plasmid encoding mCherry and the fluorescence of this protein was followed at 610 nm (excitation: 570 nm). These values at 610 nm were used to identify the portion of the OD signal at 600 nm in the co-cultures due to *E. coli* and *P. aeruginosa*. All the data are the means of three independent experiments. (**h**) Differential proteomic analyses were performed on *P. aeruginosa* and *E. coli* proteomes between co-cultures presented in panel (**d**) and those presented in panel (**a**) Heat maps: darker shades of blue indicate higher expression of the protein; darker shades of red indicate greater repression of expression of the protein. *** *p* < 0.001. For *P. aeruginosa*, FpvA and FpvB are the outer membrane transporters of ferri-PVD, FptA of ferri-PCH, PfeA and PirA of ferri-ENT, PiuA and FvbA of ferri-catechol-type siderophores, and, finally, PfeE is the esterase of the ENT pathway [25]. For *E. coli*, FepA is the outer membrane transporter of ferri-ENT, Fiu and CirA of other ferri-catechol siderophores, and FhuA and FhuE of hydroxamate siderophores.

We then co-cultured MG1655*mcherry* able to produce ENT with *P. aeruginosa* strains unable to produce PVD and PCH (∆*pvdF*∆*pchA*) and mutated for one of the genes of the ENT pathway (Figure 6). When *pfeA*, *pirA*, and *pfeS* genes were deleted, both bacterial species were able to co-exist and grow, with equivalent ODs. In contrast, when PfeE was not expressed, *E. coli* completely overtook *P. aeruginosa* within the first 20 h of culture (Figure 6d). When PfeE is not expressed, *P. aeruginosa* cannot access the iron chelated by ENT, and ENT-Fe complexes certainly accumulate in the periplasm of *P. aeruginosa* cells, as all the other proteins of the pathway (PfeA, PirA, PfeS, and PfeR) were expressed. When ∆*pvdF*∆*pchA*∆*pfeE* was grown in the presence of *E. coli* strain MG1655*mcherry*∆*entE* unable to produce ENT, *P. aeruginosa* clearly grew better than *E. coli*, confirming that sequestration of iron by ENT played a role in the shape of the growth curves of panel d in Figure 6. As iron was no longer being scavenged by ENT, *P. aeruginosa* could again access iron, even if PfeE was not expressed.

Differential proteomic analyses of *P. aeruginosa* in co-cultures between MG1655*mchery* with ∆*pvdF*∆*pchA*∆*pfeE* (Figure 6d) and MG1655*mcherry* with ∆*pvdF*∆*pchA* (Figure 6a) showed a strong difference in PfeE expression, which was due to the fact that *pfeE* was deleted in one of the conditions (∆*pvdF*∆*pchA*∆*pfeE* strain). There was no difference in PfeA expression, indicating that PfeA was expressed in equivalent proportions in *P. aeruginosa* cells in both co-cultures, even if the growth curves were completely different. We also observed no significant differences in the expression of *E. coli* outer-membrane transporters. These proteomic data indicate that differences in the shape of the growth curves of *P. aeruginosa* or *E. coli* do not necessarily involve phenotypic adaptation.

In conclusion, these data all show that PfeE plays a key and unique role in the ability of *P. aeruginosa* to acquire iron by ENT and therefore in its ability to grow in the presence of *E. coli* producing ENT, especially if *P. aeruginosa* is unable to produce its own siderophores, PVD and PCH.

## 3. Discussion

ENT is a siderophore produced by the *Enterobacteriaceae*, like *E. coli* and *Salmonella*—[4,5,6,7], but can also be produced by certain Gram-positive *Streptomyces* species [8]. In addition, many bacteria that are unable to produce ENT, such as *P. aeruginosa*, use it in a siderophore piracy strategy. As ENT is the siderophore with the highest known affinity for iron (Ka of 10^52^ M^−1^, [9]), the ability of bacteria species to use this chelator to access iron is a key asset in many microbiota.

All bacteria using ENT as a siderophore, do not contain in their genome the same gene set for the access of iron *via* this chelator. As described in the introduction, a major difference between the molecular mechanism involved in iron acquisition by ENT in *P. aeruginosa* and *E. coli* is that in *P. aeruginosa,* ENT is hydrolyzed in the bacterial periplasm, whereas in *E. coli,* it is hydrolyzed in the cytoplasm (Figure 1). *P. aeruginosa* imports ENT-Fe complexes *via* two outer-membrane transporters, PfeA and PirA [21,22,23,24] (Figure 1). If one is absent, the other can take over ENT-Fe uptake. In the periplasm, the process of iron release from ENT in *P. aeruginosa* cells involves ENT-Fe hydrolyzes by the periplasmic esterase, PfeE, into three molecules of 2,3-DHBS and an iron reduction by a yet unidentified reductase [25]. Nothing is currently known about how freed iron is imported across the inner membrane into the cytoplasm.

*P. aeruginosa* is able to detect any ENT-Fe complex in its environment using the PfeS/PfeR two-component system, and the consequence of this is an induction of the transcription of *pfeA* and *pfeE* genes [25]. This system allows a controlled phenotypic adaptation of *P. aeruginosa* to the presence of the siderophore ENT. However, in the absence of PfeS, transcription of *pfeA* and *pfeE* genes is induced, even when no ENT is present (Figure 2), suggesting that PfeR becomes constitutively active because it is no longer associated with the inner membrane sensor PfeS. The regulation of PirA transcription is also associated with a two-component system, PirS/PirR (Figure 2). However, even if ENT-Fe can be imported across the outer membrane by PirA, it is unable to activate the two-component system PirS/PirR.

In the presence of ENT, the activation of the transcription of the *pfeA* and *pfeE* genes *via* PfeS/PfeR goes hand in hand with the repression of the transcription of the genes involved in iron acquisition by the siderophore PCH, as we have already described previously (Figure 2 and [32,33]). This repression is independent of PfeS/PfeR or the expression of PfeA, PfeE or PirA. It is a consequence of the competition for iron between ENT and PCH in the bacterial environment. According to the affinities of ENT and PCH for iron (K_a_ of 10^52^ M^−1^ for ENT [9] and 10^18^ M^−2^ for PCH, [42]), most of the metal is chelated by ENT and fewer PCH-Fe complexes are formed, with the consequence that more ferric-ENT is transported into *P. aeruginosa* cells, and are available to induce their corresponding pathway. Concerning the PVD-dependent iron uptake pathway, no repression of the different genes of the PVD operon has been observed in the presence of 10 µM ENT, even if PVD has a lower iron affinity than ENT (Ka of 10^32^ M^−1^ for PVD, [37]) and ENT easily removes iron from PVD-Fe [32]. In addition, no increase in PVD production by PAO1 was observed in the presence of ENT (Figure 3) or when PAO1 was co-cultured with the *E. coli* strain *MG1655*mcherry. A large increase in PVD production was only observed for the ∆*pfeE* and ∆*pfeA*∆*pirA* mutants in the presence of ENT (Figure 3), two strains unable to access iron chelated by ENT.

Growth assays in the iron-deficient planktonic conditions of *P. aeruginosa*, alone or in co-cultures with *E. coli*, showed that the periplasmic esterase, PfeE, is a key protein in the acquisition of iron by ENT (Figure 3 and Figure 6). When absent, *P. aeruginosa* can no longer access iron *via* ENT and the presence of this siderophore in its environment becomes a major handicap. PfeA can be replaced by PirA and vice versa, but apparently no other enzyme can replace PfeE. PfeS is also irreplaceable, but its absence does not have a substantial effect on the acquisition of iron by ENT, as PfeR is constitutionally active when *pfeS* is deleted, and both PfeA and PfeE are expressed.

In co-cultures between *P. aeruginosa* and *E. coli*, *P. aeruginosa* dominates and represses the growth of *E. coli* as long as it can produce its own siderophores—PVD and PCH—or *E. coli* is unable to produce ENT. *E. coli* can only grow and co-exist with *P. aeruginosa* if two conditions are simultaneously met: *E. coli* must be able to produce ENT and *P. aeruginosa* must be deficient in the production of both of its siderophores—PVD and PCH (Figure 4). Under these conditions, both strains co-exist and share ENT to access iron. In this fragile balance of coexistence between *P. aeruginosa* and *E. coli*, with *P. aeruginosa* pirating the *E. coli* siderophore, PfeE plays a key role. If *pfeE* is deleted, *E. coli* again has the upper hand in the culture, because *P. aeruginosa* is no longer able to access the iron chelated by ENT. If *pfeA* is mutated, *E. coli* growth is less repressed than in the presence of a strain unable to express PfeE because PirA can take over the function of PfeA in iron acquisition *via* the siderophore ENT. Surprisingly, *E. coli* growth in the presence of ∆*pvdF*∆*pchA*∆*pfeA*∆*pirA* is less repressed as in the presence of a ∆*pvdF*∆*pchA*∆*pfeE* mutant. The other genes involved in the ENT pathway in *P. aeruginosa* (*pfeA*, *pfeS*, and *pfeR*) are less essential than *pfeE* for the ability of *P. aeruginosa* to grow in the presence of *E. coli*.

This battle for ferri-ENT between *P. aeruginosa* and *E. coli* involves PfeE expression but is also based on phenotypic adaptation of the expression of the various iron import pathways by *P. aeruginosa.* The presence of ENT induces the expression of PfeE and PfeA by *P. aeruginosa*, whether or not this pathogen produces PVD and/or PCH. The expression of these two proteins is absent when *E. coli* does not produce ENT. This phenotypic adaptation occurs only at the level of expression of the PfeA and PfeE proteins; the level of FpvA, FpvB, and FptA and all proteins of the PVD and PCH-dependent iron uptake pathways does not change in *P. aeruginosa*. PirA is expressed in *P. aeruginosa* in CAA medium, but its expression does not change depending on whether or not *E. coli* can produce ENT. These phenotypic adaptations are linked to the Fur regulator, which induces the expression of all proteins of the PVD- and PCH-dependent iron uptake pathways, resulting in the production of the siderophores PCH and PVD. It is also linked to the ability of ENT to induce the expression of PfeA and PfeE by *P. aeruginosa via* the PfeS/PfeR two-component system. In *E. coli*, the Fur regulator induces the transcription and expression of the ENT-dependent iron-uptake pathways under iron-restricted conditions and such activation occurs regardless of the *P. aeruginosa* mutant present in the co-culture.

Khare and Tavazoie also investigated the antagonism between *P. aeruginosa* and *E. coli* in two-species systems and highlighted the molecular complexity of the interactions [43] but were unable to identify the importance of PfeE. They showed that the siderophores PVD and PCH produced by *P. aeruginosa*, as well as the redox-active phenazines, are involved in the repression of *E. coli* growth by *P. aeruginosa* using genome-scale methods. However, they did not particularly dissect the mechanisms of the interactions concerning the iron-uptake strategies used by the two bacterial species in their battle for iron.

In conclusion, our results show that even bi-species microbial interactions are complex, including both exploitative and interference competition, for which the balance is very fragile, such that the mutation of a single gene can disrupt it. PfeE is the Achilles’ heel of *P. aeruginosa* in the presence of ENT or bacteria that can produce ENT. A strain of *P. aeruginosa* that can no longer produce PVD and PCH has the upper hand over the growth of *E. coli* as long as it can express PfeE. If *pfeE* is mutated, the growth of *P. aeruginosa* is strongly repressed by ENT-producing *E. coli*.

## 4. Materials and Methods

### 4.1. Siderophores and Growth Media

ENT, Amp (ampicillin) and Kan (kanamycin) were obtained from Sigma-Aldrich (St. Louis, MO, USA). LB broth and LB broth agar medium were purchased from Becton Dickinson (Franklin Lakes, NJ, USA).

### 4.2. Bacterial Strains, Plasmids and Growth Conditions

All the strains and plasmids used in this study are listed in Table S2 in the Supplementary Materials. *P. aeruginosa* strains were first grown in LB at 30 °C for 24 h. Afterwards, the cells were washed, resuspended and cultured overnight at 30 °C in iron-deficient casamino acid medium (CAA). The composition of CAA is 5 g L^−1^ low-iron CAA (Becton Dickinson), 1.46 g L^−1^ K_2_HPO_4_ 3H_2_O, 0.25 g L^−1^ MgSO_4_ 7H_2_O). *E. coli* strains were also first grown in LB at 30 °C for 24 h. They were then washed, resuspended and cultured overnight at 30 °C in iron-deficient CAAG medium (casamino acid medium supplemented with 0.6% glycerol). These pre-cultures were then diluted to an OD_600 nm_ of 0.01 in CAA for *P. aeruginosa* or CAAG for *E. coli* and grown at 30 °C for 24 h. MG1655mcherry needs to growth in the presence of 100 µg L^−1^ Amp and MG165*5mcherry*∆*entE* in the presence of 100 µg mL^−1^ Amp and 50 µg mL^−1^ Kan. However, for the co-cultures, no antibiotic (Amp or Kan) was added. For the co-cultures, *P. aeruginosa* and *E. coli* precultures were mixed at an OD_600 nm_ of 0.005 for each strain and grown at 30 °C in CAAG medium for 24 h.

### 4.3. Plasmid and Strain Construction

All enzymes for deoxyribonucleic acid (DNA) manipulation were purchased from ThermoFisher Scientific (Waltham, USA) and were used in accordance with the manufacturer’s instructions. The primers used are listed in Table S3 in the Supplementary Materials. The deletion mutants were constructed as described previously [24].

For the construction of the pLA48 plasmid, the cloning vector was the pGG2 plasmid expressing the DsRed sequence from the constitutive promoter of *rpsM* [44]. The insert carrying the *mcherry* sequence gene was PCR amplified from the pGBM-Kan-mCherry plasmid [45] by using the forward primer 5′-CCCCATATGGTGAGCAAGGGCGAGGAG-3′ and the reverse primer 5′-CCCAAGCTTTTACTTGTACAGCTCGTCCAT-3′. PCR products were digested using *NdeI* and *HindIII* and cloned into the pGG2 vector to generate the pLA48 plasmid expressing the mCherry sequence from the constitutive promoter of *rpsM*. The insert was verified by sequencing.

### 4.4. Quantitative Real-Time PCR

Bacteria were grown for the RT-qPCR experiments, RNA extracted and specific gene expression measured as previously described [24]. The primers used are given in Table S3. *uvrD* was used as an internal control. The transcript levels for a given gene in a given strain were normalized with respect to those for *uvrD* and are expressed as a ratio (fold change) relative to the reference conditions. *uvrD* is a housekeeping gene whose expression is stable under our experimental conditions and this gene was used to normalize the mRNA levels of genes of interest before the comparison between different samples by the real time PCR.

### 4.5. Growth of and Quantification of Fluorescence Intensity

Bacteria were grown as described above. No antibiotic was added for the co-cultures. Bacterial growth was monitored at 600 nm, mCherry fluorescence intensity at 610 nm (excitation wavelength: 570 nm) and the PVD production at 400 nm (PVD has a maximum of absorbance at 400 nm [46]).

### 4.6. Proteomics Analysis

Bacteria were grown as described above for the mono cultures and the co-cultures. Each sample for proteomic analyzes was prepared in biological triplicate. Cell pellets were prepared for proteomic analyses and the nanoLC-MS/MS dataset was obtained as described previously [32]. MS data were searched with a decoy strategy against the *P. aeruginosa* UniprotKB subdatabase (strain PAO1 reference proteome UP000002438, 5564 forward protein sequences), and the *E. coli* UniProtKB subdatabase (strain K12 reference proteome UP000000625, 4391 forward protein sequences). The data were analyzed as previously described [32]. As *P. aeruginosa* and *E. coli* can share common peptides (exactly the same amino acids sequence), we decided to exclude theses shared peptides. We used only peptides specific to *P. aeruginosa* or *E. colis* “SpecificSC”, which are peptides that do not identify any other protein in distinct protein sets in the context of the identification summaries. In the case of a *P.aeruginosa* and *E.coli* co-culture, the “SpecificSC” dataset was divided into two distinct subsets containing a specific taxonomy, each one being submitted to an independent statistical test. The size factor used to scale samples was calculated according to the DEseq2 normalization method (i.e., median of ratios method). After normalization of the data matrix, the “SpecificSC” spectral count values were submitted to a negative-binomial test using an edgeR GLM regression through R (R v3.2.5). The statistical test was based on the published msmsTests R package available in Bioconductor to process label-free LC-MS/MS data by spectral counts [47]. For each identified protein, an adjusted P-value (adjp) corrected by Benjamini–Hochberg was calculated, as well as a protein fold-change (FC). The MS data were deposited to the ProteomeXchange Consortium *via* the PRIDE partner repository with the dataset identifier PXD023712.

## Data Availability

The MS data were deposited to the ProteomeXchange Consortium via the PRIDE partner repository with the dataset identifier PXD023712.

## Supplementary Materials

The following are available online at https://www.mdpi.com/1422-0067/22/6/2814/s1. Figure S1: mCherry expression and PVD production; Figure S2: Volcano plots of the differential proteomic analyses of the *P. aeruginosa* proteome performed on the cultures and co-cultures, presented in panels a–f of Figure 4; Figure S3: Volcano plots of the differential proteomic analyses of *E. coli* proteome performed on the cultures and co-cultures, presented in panels a–f of Figure 4; Table S1: Iron uptake pathways used by *P. aeruginosa* PAO1 to access iron; Table S2: Strains and plasmids used in this study; Table S3: Oligonucleotides used in this study.
