# Supplementary material for: The Esterase PfeE, the Achilles’ Heel in the Battle for Iron between Pseudomonas aeruginosa and Escherichia coli
Source: Int J Mol Sci. 2021 Mar 10;22(6):2814. doi: 10.3390/ijms22062814 (PMC8001512; doi:10.3390/ijms22062814)
Supplement: Supplementary file 1 [file ijms-22-02814-s001.pdf]

Supplemental Material

The esterase PfeE, Achilles heel in the battle for iron between *Pseudomonas aeruginosa* and *Escherichia coli*

Véronique Gasser [1,2], Laurianne Kuhn [3], Thibaut Hubert [1,2] , Laurent Aussel [4], Philippe Hammann [3], and Isabelle J. Schalk [1,2,*]

Table S1. Iron uptake pathways used by *P. aeruginosa* PAO1 to access iron. The table gives the genes encoding TBDTs involved in iron uptake. The ligands can be siderophores (pyoverdine and pyochelin), xenosiderophores (aerobactin, rhizobactin 1021, schizokinen, vibriobactin, enterobactin, ferrichrome, mycobactin and carboxymycobactin), catecholamines, citrate or haem.

Gene	Name	Known ligand	Reference
PA4710	*phuR*	Haem	[1]
PA4675	*chtA*	Aerobactin, Rhizobactin 1021, Schizokinen	[2]
PA4514	*piuA*	catecholamines	[3]
PA4221	*fptA*	Pyochelin	[4]
PA4168	*fpvB*	Pyoverdine	[5]
PA4156	*fvbA*	Vibriobactin	[6]
PA3901	*fecA*	Citrate	[7]
PA3408	*hasR*	HasA-haem	[1]
PA2688	*pfeA*	Enterobactin	[8]
PA2466	*foxA*	Ferrioxamine	[9]
PA2398	*fpvA*	Pyoverdine	[10]
PA1910	*femA*	Mycobactins, carboxymycobactins	[11]
PA0931	*pirA*	Enterobactin,	[12,13]
PA0470	*fiuA*	Ferrichrome	[9]

Table S2. Strains and plasmids used in this study.

Strains and plasmids	Collection ID	Relevant characteristics	Reference
P. aeruginosa			
PAO1		*P. aeruginosa* wild-type strain	
ΔpfeA	PAS292	PAO1; *pfeA* chromosomally deleted	[14]
ΔpirA	PAS346	PAO1; *pirA* chromosomally deleted	This study
ΔpfeAΔpirA	PAS350	PAO1; *pfeA* and *pirA* chromosomally deleted	This study
ΔpfeE	PAS345	PAO1; *pfeE* chromosomally deleted	[14]
ΔpfeS	PAS344	PAO1; *pfeS* chromosomally deleted	This study
ΔpvdFΔpchA	PAS283	PAO1; *pvdF* and *pchA* chromosomally deleted	[15]
ΔpvdFΔpchAΔpfeA	PAS294	PAO1; *pvdF*, *pchA* and *pfeA* chromosomally deleted	[15]
ΔpvdFΔpchAΔpirA	PAS348	PAO1; *pvdF*, *pchA* and *pirA* chromosomally deleted	This study
ΔpvdFΔpchAΔpfeAΔpirA	PAS351	PAO1; *pvdF*, *pchA*, *pfeA* and *pirA* chromosomally deleted	This study
ΔpvdFΔpchAΔpfeE	PAS349	PAO1; *pvdF*, *pchA* and *pfeE* chromosomally deleted	This study
ΔpvdFΔpchAΔpfeS	PAS347	PAO1; *pvdF*, *pchA* and *pfeS* chromosomally deleted	This study
ΔpvdF	PAS263	PAO1; *pvdF* chromosomally deleted	This study
ΔpchA	PAS282	PAO1; *pchA* chromosomally deleted	This study
E. coli			
NEB5α		*fhuA2 Δ(argF-lacZ)U169 phoA glnV44 Φ80 Δ(lacZ)M15 gyrA96 recA1 relA1 endA1 thi-1 hsdR17*	New England Biolabs
SM10		thi thr leu tonA lacY supE recA::RP4-2-Tc::Mu Km λpir	[16]
MG1655		*E. coli* wild type strain	
MG1655*entE*		*entE* mutant of strain MG1655; Kan^r	[17]
MG1655*mcherry*		Derived from MG1655 strain; carrying the plamid pLA48 allowing constitutively expression of mCherry	This study
MG1655*mcherryentE*		Derived from MG1655*entE* strain; carrying the plamid pLA48 allowing constitutively expression of mCherry	This study
Plasmids			
pME3088		Suicide vector; TcR; ColE1 replicon; EcoRI KpnI DraII XhoI HindIII polylinker	[18]
pME3088*ΔpirA*	pVEGA24	pME3088 carrying the sequence to delete *pirA*	This study
pEXG2*ΔpfeE*	pVEGA23	pEXG2 carrying the sequence to delete *pfeE*	[14]
pME3088*ΔpfeS*	pVEGA22	pEXG2 carrying the sequence to delete *pfeS*	This study
pME3088*ΔpvdF*	pVEGA1	pME3088 carrying the sequence to delete *pvdF*	[15]
pME3088*ΔpchA*	pOC6	pME3088 carrying the sequence to delete *pchA*	[19]
pLA48	pLA48	Constitutive expression of mCherry	This study

Table S3. Oligonucleotides used in this study.

Oligonucleotides	Sequences (5′ to 3′)	Use
pirAatg-774F	GAGCCGGAAGCATAAATGTAAAGCAAGCTTAGCGCCTGGAGCA GGAGGTACAGGGCATGC	pVEGA24 construction
pirAatg+6R	GTAGGCGCGACCCGGCTCGTTGTAGGTTGCAGCGGCGAGATGGC CGCGACGGAATTGGGG	pVEGA24 construction
pirAstop-27F	GCAACCTACAACGAGCCGGGTCGCGCCTAC	pVEGA24 construction
pirAstop+772R	CCCGTGGAAATTAATTAAGGTACCGAATTCCCATGATGGTCCCGG TGGTGCCCATGGAGC	pVEGA24 construction
pfeSatg-777F	GAGCCGGAAGCATAAATGTAAAGCAAGCTTAGCACGCTGCGGC GCATTGGCCCATTGCCC	pVEGA22 construction
pfeSatg-6Rb	CGGCAGCCACAGGTGCAGGCACAATCCGGGCCATAACAGCGGG TGCCT GCGCATCAGGGG	pVEGA22 construction
pfeSstop-39F	CCCGGATTGTGCCTGCACCTGTGGCTGCCG	pVEGA22 construction
pfeSstop+745R	CCCGTGGAAATTAATTAAGGTACCGAATTCGTCGGTCTTGGCGAT GTT GCCGTAGACGCGG	pVEGA22 construction
uvrD F	CTACGGTAGCGAGACCTACAACAA	qRT-PCR
uvrD R	GCGGCTGACGGTATTGGA	qRT-PCR
pfeA F	GCCGAGACCAGCGTGAAC	qRT-PCR
pfeA R	GGCCGGATTCGATCTTGTT	qRT-PCR
pirA F	GCCTGAACGCTTCCCAAA	qRT-PCR
pirA R	TGAAGGCCCGTGCGATA	qRT-PCR
pfeE F	CTCGACGAGTCAACCCTGAGA	qRT-PCR
pfeE R	TAGCCGATGGCGACCAGTAG	qRT-PCR
fpvA F	AGCCGCCTACCAGGATAAGC	qRT-PCR
fpvA R	TGCCGTAATAGACGCTGGTTT	qRT-PCR
fptA F	GCGCCTGGGCTACAAGATC	qRT-PCR
fptA R	CCGTAGCGGTTGTTCCAGTT	qRT-PCR

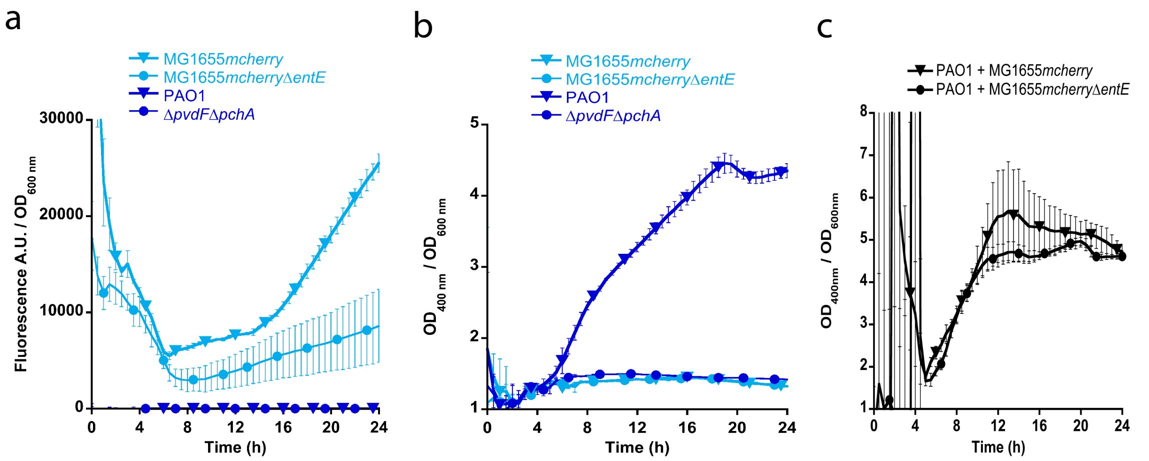

Figure S1. mCherry expression and PVD production. **a.** Emission of mCherry fluorescence corresponding to *P. aeruginosa* PAO1, Δ*pvdF*Δ*pchA*, *E. coli* MG1655*mcherry* or MG1655*mcherry*Δ*entE* strains grown alone in Figures 4a and 4b. For bacterial growth conditions, see the legend of Figure 4. Excitation wavelength: 570 nm, emission wavelength: 610 nm. **b.** PVD production by *P. aeruginosa* PAO1 and Δ*pvdF*Δ*pchA strains and E. coli* MG1655*mcherry* or MG1655*mcherry*Δ*entE* strains grown in Figures 4a and 4b. Absorbance of PVD was monitored at 400 nm and divided by the OD monitored at 600 nm. **c.** PVD production in the co-cultures between PAO1 and MG1655*mcherry* and between PAO1 and MG1655*mcherry*Δ*entE* shown in Figures 4c and 4d. Absorbance of PVD was monitored at 400 nm and divided by the OD monitored at 600 nm

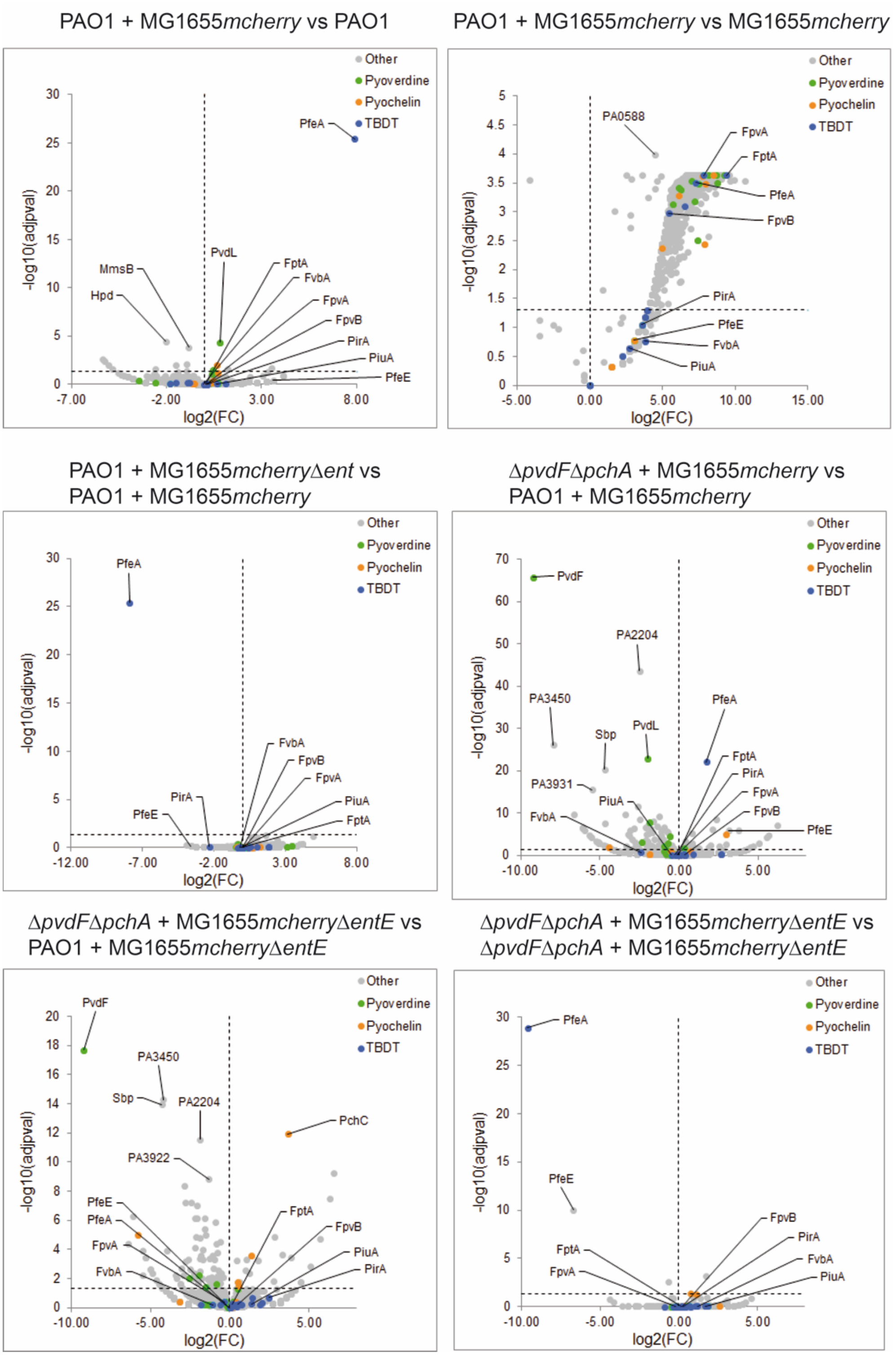

Figure S2. Volcano plots of the differential proteomic analyses of the *P. aeruginosa* proteome performed on the cultures and co-cultures presented in panels **a-f** of Figure 4. Differential proteomic analyses were performed on *P. aeruginosa* proteomes for the various combinations of co-cultures described in Figure 4c to 4f. For more details on the growth conditions, see the legend of Figure 4 and for more details on the proteomic analyses, see the Materials and Methods. The proteins of the PVD-dependent iron uptake pathway are shown in green, those of the PCH-dependent iron pathway in orange, and those of the outer-membrane transporters in blue.

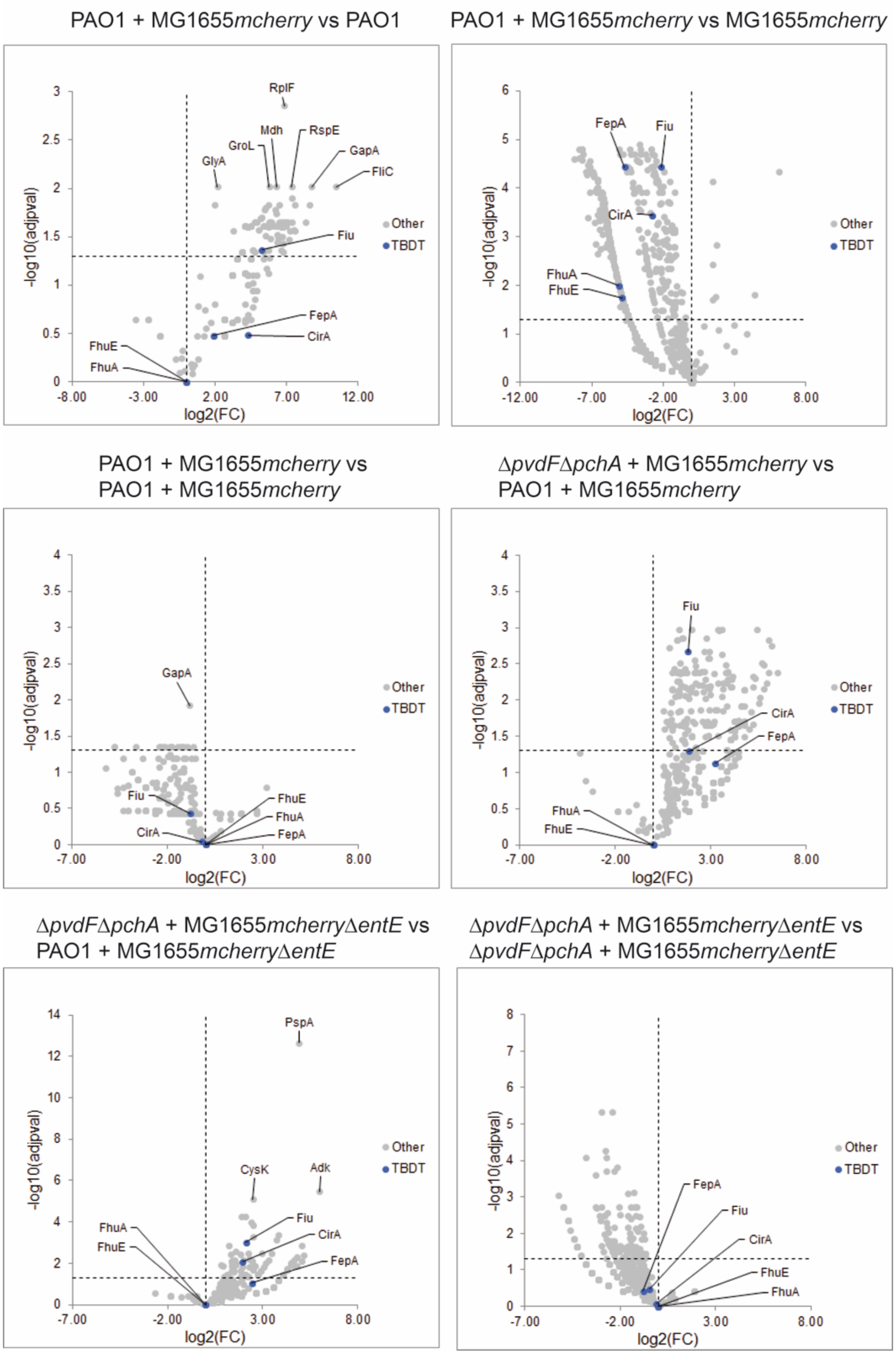

Figure S3. Volcano plots of the differential proteomic analyses of *E. coli* proteome performed on the cultures and co-cultures presented in panels **a-f** of Figure 4. Differential proteomic analyses were performed on *E. coli* proteomes for the various combinations of co-cultures described in Figure 4c to 4f. For more details on the growth conditions see the legend of Figure 4 and for more details on the proteomic analyses, see the Materials and Methods. The proteins of the PVD-dependent iron uptake pathway are shown in green, those of the PCH-dependent iron uptake pathway in orange, those of the outer-membrane transporters in blue.

REFERENCES

1. Smith, A.D.; Wilks, A. Differential contributions of the outer membrane receptors PhuR and HasR to Heme acquisition in *Pseudomonas aeruginosa*. *J. Biol. Chem.* 2015, *290*, 7756–7766, doi:10.1074/jbc.M114.633495.
2. Cuiv, P.O.; Clarke, P.; O'Connell, M. Identification and Characterization of an Iron-Regulated Gene, ChtA, Required for the utilization of the xenosiderophores aerobactin, rhizobactin 1021 and schizokinen by *Pseudomonas aeruginosa*. *Microbiology* 2006, *152*, 945–54, doi:10.1099/mic.0.28552-0.
3. Perraud, Q.; Kuhn, L.; Fritsch, S.; Graulier, G.; Gasser, V.; Normant, V.; Hammann, P.; Schalk, I.J. Opportunistic use by *Pseudomonas aeruginosa* of catecholamine neurotransmitters as siderophore to access iron. *Environ Microbiol.* 2020, doi: 0.1111/1462-2920.15372.
4. Ankenbauer, R.G.; Quan, H.N. FptA, the Fe(III)-Pyochelin receptor of *Pseudomonas aeruginosa*: A phenolate siderophore receptor homologous to hydroxamate siderophore receptors. *J Bacteriol* 1994, *176*, 307–19.
5. Ghysels, B.; Dieu, B.T.; Beatson, S.A.; Pirnay, J.P.; Ochsner, U.A.; Vasil, M.L.; Cornelis, P. FpvB, an alternative type I ferripyoverdine receptor of *Pseudomonas aeruginosa. Microbiology* 2004, *150*, 1671–80.
6. Elias, S.; Degtyar, E.; Banin, E. FvbA is required for vibriobactin utilization in *Pseudomonas aeruginosa*. *Microbiology* 2011, *157*, 2172–80, doi:10.1099/mic.0.044768-0.
7. Marshall, B.; Stintzi, A.; Gilmour, C.; Meyer, J.-M.; Poole, K. Citrate-mediated iron uptake in *Pseudomonas aeruginosa*: involvement of the citrate-inducible FecA receptor and the FeoB ferrous iron transporter. *Microbiology (Reading, Engl.)* 2009, *155*, 305–315, doi:10.1099/mic.0.023531-0.
8. Poole, K.; Young, L.; Neshat, S. Enterobactin-mediated iron transport in *Pseudomonas aeruginosa*. *J Bacteriol* 1990, *172*, 6991–6.
9. Llamas, M.A.; Sparrius, M.; Kloet, R.; Jimenez, C.R.; Vandenbroucke-Grauls, C.; Bitter, W. The heterologous siderophores ferrioxamine B and ferrichrome activate signaling pathways in *Pseudomonas aeruginosa*. *Journal of bacteriology* 2006, *188*, 1882–91.
10. Poole, K.; Neshat, S.; Krebes, K.; Heinrichs, D.E. Cloning and nucleotide sequence analysis of the ferripyoverdine receptor gene FpvA of *Pseudomonas aeruginosa*. *J Bacteriol* 1993, *175*, 4597–604.
11. Llamas, M.A.; Mooij, M.J.; Sparrius, M.; Vandenbroucke-Grauls, C.M.; Ratledge, C.; Bitter, W. Characterization of five novel *Pseudomonas aeruginosa* cell-surface signalling systems. *Molecular microbiology* 2008, *67*, 458–72.
12. Moynié, L.; Luscher, A.; Rolo, D.; Pletzer, D.; Tortajada, A.; Weingart, H.; Braun, Y.; Page, M.G.P.; Naismith, J.H.; Köhler, T. Structure and function of the PiuA and PirA siderophore-drug receptors from *Pseudomonas aeruginosa* and *Acinetobacter baumannii. Antimicrob. Agents Chemother.* 2017, *61*, doi:10.1128/AAC.02531-16.
13. Ghysels, B.; Ochsner, U.; Mollman, U.; Heinisch, L.; Vasil, M.; Cornelis, P.; Matthijs, S. The *Pseudomonas aeruginosa* PirA gene encodes a second receptor for ferrienterobactin and synthetic catecholate analogues. *FEMS microbiology letters* 2005, *246*, 167–74.

14. Perraud, Q.; Moynié, L.; Gasser, V.; Munier, M.; Godet, J.; Hoegy, F.; Mély, Y.; Mislin, G.L.A.; Naismith, J.H.; Schalk, I.J. A key role for the periplasmic PfeE esterase in iron acquisition via the siderophore enterobactin in *Pseudomonas aeruginosa*. *ACS Chem. Biol.* **2018**, *13*, 2603–2614, doi:10.1021/acschembio.8b00543.
15. Gasser, V.; Baco, E.; Cunrath, O.; August, P.S.; Perraud, Q.; Zill, N.; Schleberger, C.; Schmidt, A.; Paulen, A.; Bumann, D.; et al. Catechol siderophores repress the pyochelin pathway and activate the enterobactin pathway in *Pseudomonas aeruginosa*: an opportunity for siderophore-antibiotic conjugates development. *Environ. Microbiol.* **2016**, *18*, 819–832, doi:10.1111/1462-2920.13199.
16. Simon, R.; Priefer, U.; Puhler, A. A broad host range mobilization system for *in vivo* genetic engineering: transposon mutagenesis in gram negative bacteria. *Nat Biotech* **1983**, *1*, 784–791.
17. Martin, P.; Marcq, I.; Magistro, G.; Penary, M.; Garcie, C.; Payros, D.; Boury, M.; Olier, M.; Nougayrède, J.-P.; Audebert, M.; et al. Interplay between siderophores and colibactin genotoxin biosynthetic pathways in *Escherichia coli*. *PLOS Pathogens* **2013**, *9*, e1003437, doi:10.1371/journal.ppat.1003437.
18. Voisard, C.; Bull, C.; Keel, C.; Laville, J.; Maurhofer, M.; Schnider, U.; Défago, G.; Haas, D. Biocontrol of root diseases by *Pseudomonas fluorescens* CHAO: current concepts and experimental approaches. In *Molecular Ecology of Rhizosphere Microorganisms*; O'Gara, F., Dowling, D.N., Boesten, Eds.; VCH: Weinheim, Germany, 1994; pp. 67–89.
19. Cunrath, O.; Gasser, V.; Hoegy, F.; Reimmann, C.; Guillon, L.; Schalk, I.J. A cell biological view of the siderophore pyochelin iron uptake pathway in *Pseudomonas aeruginosa*. *Environmental microbiology* **2015**, *17*, 171–85, doi:10.1111/1462-2920.12544.
